# Nested open reading frame (ORF) 7 reverse transcription polymerase chain reaction and ORF5 phylogenetic refinement for enhanced detection and genetic classification of porcine reproductive and respiratory syndrome virus-2 in Thailand

**DOI:** 10.14202/vetworld.2025.2850-2866

**Published:** 2025-09-23

**Authors:** Tippawan Jantafong, Nipawit Karnbunchob, Wimonrat Tanomsridachchai, Pattama Mutthi, Suvarin Pavasutthipaisit

**Affiliations:** 1 Department of Preclinical Sciences, Faculty of Veterinary Medicine, Mahanakorn University of Technology, Bangkok, 10530, Thailand; 2 Department of Clinical Sciences, Faculty of Veterinary Medicine, Mahanakorn University of Technology, Bangkok, 10530, Thailand; 3 Department of Science, Technology and Innovation, Faculty of Science, Chulabhorn Royal Academy, Bangkok, 10210, Thailand; 4 Faculty of Veterinary Medicine, Rajamangala University of Technology Tawan-ok, Chonburi, 20110, Thailand; 5 Department of Pathology, Faculty of Veterinary Medicine, Mahanakorn University of Technology, Bangkok, 10530, Thailand

**Keywords:** molecular diagnostics, nested reverse transcription polymerase chain reaction, open reading frame 5 phylogeny, open reading frame 7, porcine reproductive and respiratory syndrome virus, Thailand

## Abstract

**Background and Aim:**

Porcine reproductive and respiratory syndrome virus (PRRSV) remains a major economic threat to the global swine industry, causing reproductive losses and severe respiratory illness. Accurate and cost-effective diagnostic tools are essential for timely detection and genetic monitoring, particularly in resource-limited settings. This study aimed to (i) establish a nested reverse transcription polymerase chain reaction (RT-PCR) assay targeting the open reading frame 7 (ORF7) gene to detect and differentiate PRRSV-1 and PRRSV-2, and (ii) refine the genetic classification of PRRSV-2 strains circulating in Thailand through ORF5-based phylogenetic analysis.

**Materials and Methods:**

A nested RT-PCR assay was developed using external primers for general PRRSV detection and internal primers specific to PRRSV-1 and PRRSV-2. Analytical specificity was assessed against modified-live vaccines, clinical isolates, and heterologous swine viruses (swine influenza virus and foot-and-mouth disease virus). Diagnostic accuracy was evaluated using 96 clinical serum samples and compared with a commercial real-time RT-PCR kit. To confirm genotyping capability, ORF7-positive samples underwent ORF5 sequencing and phylogenetic analysis. In addition, 386 complete ORF5 sequences (2000–2023) from Thai isolates and global references were analyzed using maximum likelihood methods to refine lineage and sublineage classification.

**Results:**

The nested ORF7 RT-PCR assay demonstrated high specificity without cross-amplification and achieved 100% concordance with real-time RT-PCR, confirming its diagnostic reliability. Among the clinical samples, PRRSV-1, PRRSV-2, and mixed infections were successfully detected. Sequencing confirmed strain identities and revealed close similarity with both endemic and vaccine-related strains. Phylogenetic analysis classified Thai PRRSV-2 strains into five lineages (L1, L5, L8, L9, L10) and five sublineages (L1I, L5A, L8C, L8E, L9D). Notably, this study is the first to report sublineages L8C and L9D in Thailand, while also documenting a lineage shift from L8E to L10 as the predominant circulating strain.

**Conclusion:**

The integration of nested ORF7 RT-PCR with ORF5-based phylogenetic analysis provides a sensitive, affordable, and reliable diagnostic platform for PRRSV detection and genetic classification. These findings enhance understanding of PRRSV-2 diversity in Thailand, highlight emerging sublineages, and underscore the importance of continuous molecular surveillance to inform vaccine strategies and disease control policies.

## INTRODUCTION

The porcine reproductive and respiratory syndrome virus (PRRSV) is the causative agent of porcine reproductive and respiratory syndrome (PRRS), a disease that leads to severe reproductive disorders in sows, late-term abortions, and respiratory distress in piglets [1]. These clinical effects contribute to reduced productivity, increased mortality in suckling and weaned pigs, and substantial economic losses to the swine industry [1]. PRRSV is a small, enveloped virus with a positive-sense, single-stranded RNA genome of approximately 15 kb. It is classified within the order *Nidovirales*, family *Arteriviridae*, and genus *Porartevirus* [2]. The genome comprises 11 open reading frames (ORFs). ORF1a and ORF1b encode nonstructural proteins critical for viral replication, whereas ORFs 2–7 encode structural proteins, including glycoproteins 2–5 (GP2–GP5), the membrane (M) protein, and the nucleocapsid (N) protein [3–5]. The N protein, encoded by ORF7, is the most abundant structural component and is essential for viral assembly, replication, and modulation of the host immune response [6, 7]. Its high intracellular expression increases the sensitivity of diagnostic assays targeting ORF7, particularly in low viral load samples. Although ORF7 is relatively conserved within each genotype, its sequence variation between genotypes and lineages makes it a suitable target for both sensitive detection and genotypic differentiation [5, 8, 9]. Compared with highly variable regions such as ORF5, ORF7 provides a more conserved and stable diagnostic target, making it especially advantageous for nested reverse transcription polymerase chain reaction (RT-PCR) assays designed to reliably detect and differentiate PRRSV strains.

PRRSV is divided into two species, PRRSV-1 and PRRSV-2, based on antigenic and genetic properties, both of which are widely distributed in swine populations worldwide [2]. Differentiation between the two species typically relies on sequence analyses of ORF5 and ORF7. ORF5, which encodes the major envelope protein GP5, is the most variable structural gene, allowing detailed phylogenetic resolution. Despite its variability, which can lower sensitivity for direct detection, ORF5 remains indispensable for evolutionary studies and lineage classification. By contrast, ORF7, encoding the abundantly expressed N protein with relatively low mutation rates, is conserved within species but divergent between PRRSV-1 and PRRSV-2, thus serving as a reliable marker for species-level differentiation [5]. Phylogenetic studies based on ORF5 have categorized PRRSV-1 into four subtypes, with subtype 1 (Global) containing 12 clades (A–L), and PRRSV-2 into 11 lineages further divided into 21 sublineages [10–14]. Although PRRSV has circulated in Thailand for more than three decades, predominantly PRRSV-2 [15], detailed genetic characterization of PRRSV-2 remains limited, emphasizing the need for improved molecular tools to strengthen detection and classification.

Accurate and rapid PRRSV detection is crucial for effective disease management. Quantitative RT-PCR (qRT-PCR) is considered the gold standard due to its high sensitivity, specificity, and ability to quantify viral loads [16]. However, qRT-PCR requires expensive instruments, specialized reagents, and trained personnel, limiting its utility in resource-limited laboratories. Conventional RT-PCR offers a more cost-effective alternative with acceptable sensitivity and specificity, but it performs poorly in samples with low viral loads and is more susceptible to non-specific amplification. Nested RT-PCR, which involves two rounds of amplification with external and internal primer sets, significantly enhances both sensitivity and specificity. This method improves detection of low-copy viral RNA and reduces false negatives, making it a practical, straightforward, and reliable option for laboratories lacking advanced molecular infrastructure [17].

Although several molecular techniques such as RT-PCR, qRT-PCR, loop-mediated isothermal amplification (LAMP), and sequencing have been employed for detecting and differentiating PRRSV, each method has practical limitations in routine diagnostics. qRT-PCR, while highly sensitive, requires costly equipment and technical expertise, restricting its accessibility in resource-limited laboratories. LAMP assays are simpler but prone to false positives, whereas sequencing and next-generation methods, though powerful, are not feasible for widespread field application due to cost and complexity. Importantly, the genetic characterization of PRRSV-2 in Thailand has not been comprehensively updated in recent years, despite the virus being endemic for over three decades. Previous studies primarily focused on limited regions or short timeframes, leaving gaps in understanding the full genetic landscape, lineage shifts, and emergence of novel sublineages. Furthermore, there is a lack of affordable, highly specific diagnostic assays that can both differentiate PRRSV genotypes and support genetic monitoring to guide vaccine and control strategies. This gap underscores the urgent need for diagnostic tools that combine sensitivity, specificity, and cost-effectiveness, while simultaneously contributing to refined phylogenetic classification of PRRSV-2 strains in Thailand.

This study was designed to address these diagnostic and epidemiological gaps by developing and optimizing a nested ORF7 RT-PCR assay for the detection and differentiation of PRRSV-1 and PRRSV-2. Specifically, the objectives were to: (i) Establish and validate the analytical specificity and diagnostic accuracy of the assay using field samples, vaccines, and heterologous swine viruses; and (ii) refine the genetic classification of Thai PRRSV-2 strains through ORF5-based phylogenetic analysis using both recent clinical isolates and a comprehensive dataset of historical sequences. By integrating a cost-effective molecular diagnostic tool with large-scale genetic surveillance, this study aims to provide a robust platform for improving routine diagnosis, strengthening molecular epidemiology, and informing vaccine development and disease control strategies in Thailand and other resource-limited settings.

## MATERIALS AND METHODS

### Ethical approval

All procedures involving animals were reviewed and approved by the Institutional Animal Care and Use Committee of the Faculty of Veterinary Medicine, Mahanakorn University of Technology, Thailand (Approval number: ACUC-MUT-010). Sample collection and handling complied with established institutional and national guidelines for animal welfare.

Moreover, all virus manipulation and molecular diagnosis were performed in accordance with institutional guidelines on biosafety and standard procedures for handling potentially infectious biological materials. All samples were processed at biosafety level 2. All staff who worked on possibly infectious samples wore appropriate PPE within containment laboratories. The decontaminated waste was disposed of according to the institutional biosafety protocols.

### Study period and location

This study was conducted from January 2023 to February 2024 at the Faculty of Veterinary Medicine, Mahanakorn University of Technology, Thailand. Serum samples were collected from three commercial pig farms in the eastern and central regions of Thailand from May to December 2023. These farms were located in Bo Thong District, Chonburi Province; Bang Khla District, Chachoengsao Province; and Samchuk District, Suphanburi Province. Farm selection was based on previous reports of PRRSV outbreaks and their geographic representation of Thailand’s swine industry.

### Clinical sample collection

A total of 96 serum samples were collected from fattening pigs across the three selected farms, each of which housed approximately 1,000 animals. Pigs were selected for blood collection within each farm using a simple random sampling method, irrespective of clinical signs, to reduce sampling bias. Trained veterinary personnel collected blood samples under aseptic conditions. Serum was separated by centrifugation and stored at –80°C until RNA extraction.

### Reference viruses and vaccines

In this study, we used three PRRSV-modified live virus (MLV) vaccines and three clinically positive samples as virus templates for the specificity test. The clinically positive samples were stored in our laboratory. The positive specificity test included Suvaxyn PRRS (Zoetis, USA), a vaccine against PRRSV-1 (subtype 1) strain 96V198, with a viral load of 10^5.2^ TCID_50_; Unistrain PRRS (Hipra, Spain), a vaccine against PRRSV-1 (subtype 1) strain VP-046 BIS, with a viral load of 10^5.2^ TCID_50_; Fostera PRRS (Zoetis) PRRSV-2, a vaccine against PRRSV-2 (Lineage 8), with a viral load of 10^3.1^ TCID_50_; highly pathogenic (HP) PRRSV was represented by a clinically positive sample strain HP-PRRSV-MUT-2010, with a viral load of 10^4.5^ TCID_50_. Negative specificity tests included clinically positive samples of swine influenza virus (SIV) and foot-and-mouth disease virus (FMDV).

### Primer selection, RNA extraction, complementary DNA (cDNA) synthesis, and RT-PCR

The primers used in this study comprised two sets: an external primer and an internal primer. The external primer (PRRSV-UF/UR), drawn from the research of Oleksiewicz *et al*. [18], is specific to both virus strains (PRRSV-1 and PRRSV-2) and was used to confirm PRRSV infection. The internal primer consists of two pairs of primers, each specific to the ORF7 gene of a particular virus strain. The primer set PRRSV1-F/R targets PRRSV-1, while the primer set US-F/R targets PRRSV-2, aiding in PRRSV classification. These primer sets were selected from Theveethivarak *et al*. [19] and Chen *et al*. [20], respectively (Table 1).

**Table 1:** Primers used in the nested ORF7 RT-PCR.

Primer set	Primer	Sequences (5’–3’)	Target gene	Product size (bp)
External primer	PRRSV-UF	GCCCCTGCCCAYCACG	ORF7	PRRSV-1: 637
	PRRSV-UR	TCGCCCTAATTGAATAGGTGA		PRRSV-2: 660
Internal primer	PRRSV1-F	CAGGACTTCGGAGCCTCGT	ORF7	PRRSV-1: 158
	PRRSV1-R	AGCAACTGGCACAGTTGATTGA		
	US-F	CATCGCTCAGCAAAACCA	ORF7	PRRSV-2: 287
	US-R	CATCATGCTGAGGGTGATGCT		

RT-PCR = Reverse transcription polymerase chain reaction, PRRSV = Porcine reproductive and respiratory syndrome virus, ORF = Open reading frame, UF = Universal forward primer, UR = Universal reverse primer

Total RNA was extracted from virus templates (clinically positive samples), vaccine templates, and serum samples obtained from pig-fattening farms using a viral RNA extraction kit (Geneaid, Taiwan) according to the manufacturer’s instructions. Subsequently, the isolated RNA was converted into cDNA using random hexamer primers and M-MuLV Reverse Transcriptase (Vivantis Technologies, Malaysia) according to the manufacturer’s instructions. The cDNA synthesis involved incubation at 42°C for 60 min, followed by heating at 85°C for 5 min. This process was carried out using a reaction mixture comprising 8 μL of RNA, 1 μL of random hexamers, 1 μL of dNTP, 7 μL of RNase-free H_2_O, 2 μL of 10X Buffer M-MuLV, and 1 μL of M-MuLV Reverse Transcriptase. The resulting cDNA is stored at −20°C until further testing.

The efficiency of each primer pair (Table 1) was tested using the single RT-PCR method. Furthermore, the appropriate annealing temperature for each pair of primers was determined. A total volume of 20 μL of RT-PCR reaction was prepared, consisting of 14.12 μL distilled H_2_O, 2 μL 10X PCR Buffer, 0.6 μL 50 mM MgCl_2_, 0.4 μL 10 mM dNTP mix, 0.4 μL 10 pM forward primer, 0.4 μL 10 pM reverse primer, 0.08 μL Platinum *Taq* DNA Polymerase (Invitrogen, USA), and 2 μL cDNA. RT-PCR was conducted under the following conditions in a thermal cycle: initial denaturation at 94°C for 2 min, followed by 34 cycles of denaturation at 94°C for 30 s, annealing at 54°C–58°C for 40 s, elongation at 72°C for 40 s, and final elongation at 72°C for 7 min. The amplified products were then subjected to electrophoresis in a 1.2% agarose gel in Tris-acetate-EDTA (1X TAE) solution, at a constant voltage of 50 V for 50 min. Subsequently, the gel was stained with SafeView Classic dye (Abm, Canada) to visualize the DNA bands and examined under a dark reader blue light transilluminator (Major Science, Taiwan).

### Establishment of the nested ORF7 RT-PCR

The optimized nested ORF7 RT-PCR requires adjusting the chemicals and the optimized first-round PCR product, which serves as the DNA template for the second round, to ensure efficient amplification. The adjusted parameters included the volume of the first-round PCR product, the annealing temperature and time, and the extension time. Each nested RT-PCR reaction was conducted in a total volume of 10 μL, composed of 1 μL of 10X PCR Buffer, 0.3 μL of 50 mM MgCl_2_, 0.2 μL of 10 mM dNTP mix, 0.2 μL of 10 pM forward primer, 0.2 μL of 10 pM reverse primer, 0.04 μL of Platinum *Taq* DNA Polymerase (Invitrogen), and the second-round DNA template in amounts adjusted to 1, 0.5, or 0.2 μL. Sterilized distilled water (dH_2_O) was added to obtain the final reaction volume. The second-round PCR cycles were optimized by adjusting the annealing temperature (from 54°C to 62°C), annealing time (from 10 s to 40 s), and extension time (from 15 s to 45 s) to minimize non-specific bands in the PCR product. PCR products were analyzed through electrophoresis on a 1.2% agarose gel in Tris-acetate-EDTA (1X TAE) buffer, using a constant voltage of 50 V for 50 min. After electrophoresis, the gel was stained with SafeView Classic dye (Abm) to visualize the DNA bands, which were then examined under a Dark Reader blue light transilluminator (Major Science, Taiwan).

### Preliminary diagnostic performance evaluation of the nested ORF7 RT-PCR assay

#### Specificity of nested ORF7 RT-PCR

The positive specificity test of nested ORF7 RT-PCR was conducted to confirm that both pairs of internal primers, PRRSV1-F/R (158 base pairs [bp]) and US-F/R (287 bp), were specific to each virus strain and would not result in cross-amplification when testing with the mixed-virus template. The viruses and vaccine templates used in the analytical positive specificity analysis included the PRRSV-1 Unistrain vaccine, PRRSV-1 Suvaxyn vaccine, PRRSV-2 Fostera vaccine, and clinically positive samples. These clinically positive samples included HP-PRRSV-MUT-2010 (PRRSV-2; HP-PRRSV), PRRSV/RBR5/2022 (sample infected with both PRRSV strains), PRRSV/RBR6/2022 (sample infected with PRRSV-1), and PRRSV/RBR8/2022 (sample infected with PRRSV-2). The negative specificity test was performed using other commonly found viruses in pigs, including SIV and FMDV, which served as clinically positive samples.

#### Diagnostic accuracy evaluation of nested ORF7 RT-PCR

Ninety-six serum samples collected from fattening pigs were used to assess the preliminary diagnostic performance of ORF7 nested RT-PCR. These samples were tested using the established ORF7 nested RT-PCR method to detect and differentiate PRRSV. Subsequently, the positive samples were forwarded to a private animal disease diagnosis laboratory for confirmation using a commercial PRRSV screening real-time RT-PCR kit, ID Gene PRRSV Triplex (LABGENE Scientific SA, Switzerland). This commercial real-time RT-PCR, which can simultaneously detect and differentiate PRRSV-1 and PRRSV-2, serves as a reference PCR in this study. The consistency of the experimental results between the ORF7 nested RT-PCR and the real-time RT-PCR was assessed using the concordance rate percentage (percentage agreement) to evaluate the diagnostic accuracy of the clinical samples. The formula of the calculated concordance rate is as follows:


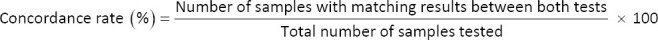


### ORF5 amplification and sequencing

To confirm the nested ORF7 RT-PCR results and determine the PRRSV genotypes, we selected seven samples that showed strong positive reactions for ORF5 gene amplification and sequencing. This additional molecular characterization was performed to confirm the genotyping capability of the nested ORF7 assay and provide comprehensive strain identification. ORF5 RT-PCR was performed using two pairs of ORF5-specific primers as described by Jantafong *et al*. [21]. The primer sets used to distinguish between PRRSV genotypes were as follows: PRRSV-1-specific primers (forward: CAATGAGGTGGGCIACAACC; reverse: TATGTIATGCTAAAGGCTAGCAC) and PRRSV-2-specific primers (forward: CAGGCATCGTGGCTGTGTG; reverse: TCAAAAGGTGCAGAAGCCCT). The PCR product sizes for PRRSV-1 and PRRSV-2 were 719 and 915 bp, respectively. Using a cDNA template, 10 pmol of each genotype-specific primer, 10× *Taq* polymerase buffer, 10 mM dNTP, 25 mM MgCl_2_, and 2.5 units of Platinum *Taq* DNA polymerase (Invitrogen), RT-PCR amplification was performed in a final volume of 100 μL. The PCR cycle methodology comprised initial denaturation at 94°C for 3 min, 40 cycles of denaturation at 94°C for 30 s, annealing at 56°C for 40 s, and elongation at 72°C for 1 min, with a final elongation step at 72°C for 10 min. A Gel/PCR Fragment Extraction Kit (RBC Bioscience, Taiwan) was used to purify the PCR products after they were amplified. Subsequently, the purified amplicons were submitted to Macrogen, a commercial sequencing facility in Korea, for bidirectional sequencing using both forward and reverse primers.

The complete ORF5 DNA sequences were assembled using the Segman tool in Lasergene Molecular Biology software (version 17.1; DNASTAR, Madison, WI, USA). The percentage identities of the seven PRRSV strains analyzed in this study were compared with reference sequences representing PRRSV-1 and PRRSV-2 from the GenBank database using the Nucleotide Basic Local Alignment Search Tool (BLAST) (https://blast.ncbi.nlm.nih.gov/Blast.cgi).

### Phylogenetic analysis of PRRSV-1 and PRRSV-2

To confirm genetic classification, phylogenetic trees were constructed using 205 PRRSV sequences, with all sequences trimmed to obtain the full-length ORF5 gene (PRRSV-1: 606 bp and PRRSV-2: 603 bp). The dataset comprised 87 PRRSV-1 sequences, including 84 PRRSV-1 reference sequences and 3 ORF5 PRRSV-1 sequences. Similarly, 118 PRRSV-2 sequences were analyzed, including 114 PRRSV-2 reference sequences and 4 ORF5 PRRSV-2 sequences. Subsequently, the phylogenetic trees of the PRRSV-1 and PRRSV-2 sequences were analyzed separately for clarity. The phylogenetic trees were constructed using MEGA X software version 10.2.6 [22]. Multiple sequence alignments were performed using the ClustalW algorithm combined with MEGA X, followed by phylogenetic analysis employing the Kimura 2-parameter model’s maximum likelihood method. To ensure the robustness of the analysis, 1,000 bootstrap replicates were included. The complete ORF5 gene sequences from this study are available in the GenBank database under accession numbers PV440586–PV440592. Details of all PRRSV sequences used in this study are provided in supplementary data (Table S1).

### Refined genetic classification of PRRSV-2 strains in Thailand

#### PRRSV-2 sequence datasets

All available ORF5 sequences deposited in the GenBank database from 2000 to 2023 were retrieved to investigate the genetic diversity of PRRSV-2 in Thailand. Sequences with 100% nucleotide identity, those exhibiting high similarity and close phylogenetic relatedness, and incomplete ORF5 sequences were systematically excluded from the analysis to ensure data quality and reliability. A comprehensive phylogenetic analysis was conducted using this curated dataset to refine the genetic classification of PRRSV-2 strains in Thailand.

Based on the updated genetic classification system for PRRSV-2 lineages [13], the virus is categorized into lineages L1 (sublineages L1A–L1F and L1H–L1J), L2, L3, L4, L5 (L5A–L5B), L6, L7, L8 (L8A–L8E), L9 (L9A–L9E), L10, and L11. The reference sequences used in this study were randomly selected from a curated pool of 1,100 lineage and sublineage reference sequences identified in previous studies. The selected sequences (excluding L2 and L11) served as representative examples for each lineage and sublineage analyzed in this study. This analysis utilized 386 complete ORF5 gene sequences obtained from the GenBank database, which were organized into three distinct categories: (1) 190 representative ORF5 sequences from Thai PRRSV-2 isolates; (2) 187 reference sequences originating from China, Taiwan, South Korea, Japan, Vietnam, Canada, Mexico, and the USA; and (3) 9 sequences derived from PRRSV-2 vaccine strains (supplementary data Table S2). Subsequently, the phylogenetic trees of the 138 ORF5 PRRSV-2 sequences belonging to lineage L1 were independently analyzed to gain a deeper understanding of the sublineages within L1 in the context of Thailand. The detailed data, including accession numbers, genotypes, collection locations, and dates, were systematically compiled and summarized in the supporting information (Table S2) to ensure the study’s transparency and reproducibility.

#### Phylogenetic analysis of the Thai PRRSV-2 strain

To refine the genetic classification of Thai PRRSV-2 strains, all sequences were trimmed to obtain full-length ORF5 sequences (603 bp). Multiple sequence alignments were performed using the ClustalW algorithm in MEGA X software version 10.2.6 [22]. Phylogenetic analysis was conducted employing the maximum likelihood method based on the Kimura 2-parameter model. To ensure robustness of the analysis, bootstrap values were calculated from 1,000 replicates to assess branch support.

## RESULTS

### Evaluation of a single RT-PCR for the detection of PRRSV-1 and PRRSV-2

**Figure 1:**
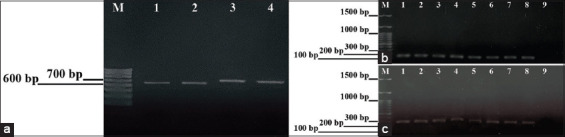
The efficacy and specificity of the primers. (a) Test of each external primer pair for the detection of PRRSV-1 and PRRSV-2 using a single RT-PCR. The specific amplicon bands of PRRSV-1 (637 bp) and PRRSV-2 (660 bp) are shown on an agarose gel as follows: Lane M: DNA ladder (1000 bp); lane 1: PRRSV-1 Unistrain vaccine; lane 2: Suvaxyn vaccine; lane 3: PRRSV-2 Fostera vaccine; lane 4: HP-PRRSV-MUT-2010. (b) Test of each internal primer pair for detection of PRRSV-1 (Suvaxyn vaccine) is shown as follows: Lane M: DNA ladder (1000 bp); lane 1–8: RT-PCR products of different annealing temperatures condition (62°C, 61.4°C, 60.4°C, 58.9°C, 57.1°C, 55.5°C, 54.6°C, 54°C, respectively); lane 9: Negative control. The PRRSV-1 product was amplified as a band of 158 bp. (c) Test of each internal primer pair for detection of PRRSV-2 (Fostera vaccine) using a single RT-PCR is shown as follows: Lane M: DNA ladder (1000 bp); lanes 1–8: Specific amplicon bands are visualized corresponding to different annealing temperatures (62°C, 61.4°C, 60.4°C, 58.9°C, 57.1°C, 55.5°C, 54.6°C, 54°C, respectively); lane 9: Negative control. The 287-bp amplicon bands are specific for PRRSV-2. RT-PCR = Reverse transcription polymerase chain reaction, PRRSV = Porcine reproductive and respiratory syndrome virus, ORF = Open reading frame.

The performance of each primer pair in detecting PRRSV-1 and PRRSV-2 (PRRSV-1 Unistrain vaccine, PRRSV-1 Suvaxyn vaccine, PRRSV-2 Fostera vaccine, HP-PRRSV-MUT-2010) was assessed using a single RT-PCR assay to verify reaction specificity. Figure 1 illustrates the RT-PCR products obtained under optimal annealing conditions, demonstrating no cross-reactivity. The specific amplicon bands generated by each external primer pair on agarose gel are shown in Figure 1a. At an annealing temperature of 58°C, the expected amplicon sizes were 637 bp for PRRSV-1 and 660 bp for PRRSV-2. Subsequently, the annealing temperatures of the internal primer pairs were optimized for PRRSV-1 (Suvaxyn vaccine; Figure 1b) and PRRSV-2 (Fostera vaccine; Figure 1c), tested at 62°C, 61.4°C, 60.4°C, 58.9°C, 57.1°C, 55.5°C, 54.6°C, and 54°C. The resulting RT-PCR products were characterized by amplicon sizes of 158 bp (PRRSV-1) and 287 bp (PRRSV-2). Agarose gel electrophoresis confirmed that the optimal annealing temperature for external primers was 58°C, while for internal primers, it was 54°C.

### Optimal conditions of nested ORF7 RT-PCR

The optimal conditions for the nested ORF7 RT-PCR were determined through systematic optimization of chemical composition and thermal cycling parameters. The ratios between the master mix and cDNA template were refined and standardized for both the first and second rounds of nested RT-PCR (Table 2). In addition, the cycling protocol was optimized by adjusting annealing temperature, annealing time, and extension duration, with the final parameters summarized in Table 3. These adjustments enhanced amplification efficiency and specificity, consistently producing clear bands of the expected sizes for PRRSV-1 (158 bp) and PRRSV-2 (287 bp), while minimizing non-specific amplification.

**Table 2:** Details of the optimal conditions of the nested ORF7 RT-PCR reaction mixture.

Component	Nested RT-PCR (Volume [μL])	

	1^st^ round	2^nd^ round
10X PCR buffer	1	1
50 mM MgCl_2_	0.3	0.3
10 mM dNTP mix	0.2	0.2
10 pM forward primer	0.2 (PRRSV-UF)	0.2 (PRRSV1-F or US-F)
10 pM of reverse primer	0.2 (PRRSV-UR)	0.2 (PRRSV1-R or US-R)
TaqDNA polymerase	0.04	0.04
cDNA template (each sample)	1	0.5
Sterilized distilled water	Up to final volume 10 μL	Up to final volume 10 μL

RT-PCR = Reverse transcription polymerase chain reaction, PRRSV = Porcine reproductive and respiratory syndrome virus, ORF = Open reading frame, UF = Universal forward primer, UR = Universal reverse primer, US-F = PRRSV-2 forward primer, US-R = PRRSV-2 reverse primer

**Table 3:** Summary of the nested ORF7 RT-PCR cycling used for the detection of PRRSV-1 and PRRSV-2.

RT-PCR cycle	Nested RT-PCR protocol				Cycles

	1^st^ round		2^nd^ round		
	
	Temperature (°C)	Time	Temperature (°C)	Time	
Initial denaturation	94	2 min	94	2 min	1
Denaturation	94	30 s	94	30 s	34
Annealing	58	40 s	61	10 s	
Extension	72	40 s	72	25 s	
Final extension	72	7 min	72	5 min	1

RT-PCR = Reverse transcription polymerase chain reaction, PRRSV = Porcine reproductive and respiratory syndrome virus, ORF = Open reading frame

### Specificity test of nested ORF7 RT-PCR

**Figure 2:**
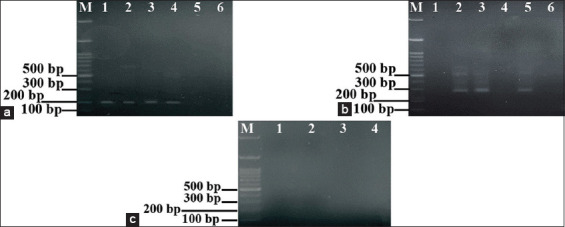
Specificity test of nested ORF7 RT-PCR. (a) PRRSV-1 detection using PRRSV1-F/R internal primers (158 bp). Lane M: DNA ladder (1000 bp); lane 1: PRRSV-1 Unistrain vaccine; lane 2: PRRSV-1 Suvaxyn vaccine; lane 3: The mixed-virus (PRRSV-1 and PRRSV-2) positive sample (PRRSV/RBR5/2022) was specifically amplified for PRRSV-1 in a 158 bp amplicon; lane 4: PRRSV/RBR6/2022 (PRRSV-1); lane 5: PRRSV/RBR8/2022 (PRRSV-2); lane 6: Negative control. (b) PRRSV-2 detection using internal primers (US-F/R, 287 bp). Lane M: DNA ladder (1000 bp); lane 1: PRRSV-1 Unistrain vaccine; lane 2: PRRSV-2 Fostera vaccine; lane 3: the mixed-virus (PRRSV-1 and PRRSV-2) positive sample (PRRSV/RBR5/2022) was specifically amplified for PRRSV-2 in a 287 bp amplicon; lane 4: PRRSV/RBR6/2022 (PRRSV-1); lane 5: PRRSV/RBR8/2022 (PRRSV-2); lane 6: negative control. (c) Negative specificity test shows the absence of amplification in non-target viruses. Lane M: DNA ladder (1000 bp); lane 1: SIV-positive sample tested with PRRSV1-F/R primers; lane 2: SIV-positive sample tested with US-F/R primers; lane 3: FMDV-positive sample tested with PRRSV1-F/R primers; lane 4: FMDV-positive sample tested with US-F/R primers. RT-PCR = Reverse transcription polymerase chain reaction, PRRSV = Porcine reproductive and respiratory syndrome virus, ORF = Open reading frame.

Specific nested RT-PCR products were visualized by agarose gel electrophoresis. These products were distinctly amplified in separate master-mix tubes using internal primers PRRSV1-F/R (Figure 2a) and US-F/R (Figure 2b). The assay demonstrated no cross-amplification in a mixed-virus template (PRRSV/RBR5/2022; Lane 3, Figure 2a and b) and no cross-reactivity with other viruses such as SIV and FMDV (Figure 2c). The optimized first-round nested RT-PCR generated sufficient PCR products for PRRSV detection, and the refined second-round assay further improved the detection of specific PRRSV strains. The specificity was confirmed by the presence of a 158-bp band for PRRSV-1 in lanes 1, 2, and 4 (Figure 2a) and a 287-bp band for PRRSV-2 in lanes 2 and 5 (Figure 2b). These results confirm that the nested ORF7 RT-PCR reaction mixture and protocol were effectively developed for specific detection and differentiation of PRRSV-1 and PRRSV-2.

### Diagnostic accuracy of nested ORF7 RT-PCR

**Figure 3:**
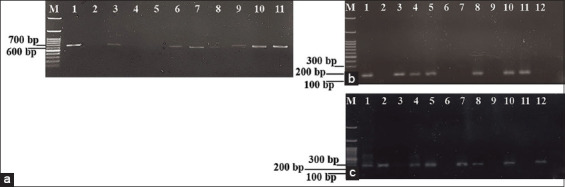
Representative agarose gel electrophoresis images of nested ORF7 RT-PCR for the detection of PRRSV-1 and PRRSV-2 from clinically swine serum samples. (a) The first-round nested RT-PCR using external primers for PRRSV-1 (637 bp) and PRRSV-2 (660 bp). Lane M: DNA ladder (1000 bp); lane 1: Positive control; lanes 2–11: the amplicons from samples number: PRRSV/MUT52, -53, -54, -55, -56, -57, -58, -59, -60, and -61, respectively. (b) Second-round nested RT-PCR using PRRSV1-F/R internal primers specific for PRRSV-1. Lane M: DNA ladder (1000 bp); lane 1: Positive control for PRRSV-1 (158 bp); lanes 2–12: The amplicons from samples as follows: PRRSV/MUT46, -47, -49, -50, -53, -56, -57, -58, -59, -60, and -61. (c) The second-round nested RT-PCR using internal primers US-F/R, specific for PRRSV-2. Lane M: DNA ladder (1000 bp); lane 1: Positive control for PRRSV-2 (287 bp); lanes 2–12: The amplicons correspond to sample numbers: PRRSV/MUT46, -47, -49, -50, -53, -56, -57, -58, -59, -60, and -61, respectively. RT-PCR = Reverse transcription polymerase chain reaction, PRRSV = Porcine reproductive and respiratory syndrome virus, ORF = Open reading frame, MUT46 = Sample ID used in this study.

The diagnostic performance of the nested ORF7 RT-PCR was evaluated using 96 swine serum samples. Of these, 24 samples (25%, n = 24/96) displayed amplicon bands of PRRSV-1 (637 bp) or PRRSV-2 (660 bp) when amplified with the external primers PRRSV-UF/UR (Figure 3a). All 24 positive samples were subsequently analyzed by the second-round nested ORF7 RT-PCR, which successfully distinguished PRRSV-1 (158 bp) from PRRSV-2 (287 bp). The results showed that 3 samples (12.50%, n = 3/24) were positive for PRRSV-1, 14 samples (58.33%, n = 14/24) for PRRSV-2, and 4 samples (16.67%, n = 4/24) were co-infected (Figure 3b and c). Three samples that tested positive in the first round were not amplified in the second round and were excluded from subsequent real-time RT-PCR validation. Importantly, the nested RT-PCR results exhibited complete concordance with real-time RT-PCR, confirming the reliability of the developed method. Table 4 summarizes all positive PRRSV samples identified by nested ORF7 RT-PCR. Collectively, these findings demonstrate that the established nested ORF7 RT-PCR is a practical, accurate, and effective diagnostic tool for detecting PRRSV infection in swine herds in Thailand.

**Table 4:** Details of the ORF7 nested RT-PCR results of PRRSV-1 and PRRSV-2 detection from clinically positive swine serum samples.

No.	Sample number	Nested ORF7 RT-PCR results			Concordance rate (%) (real-time RT-PCR)

		1^st^ round^a^	2^nd^ round^b^		

			PRRSV-1	PRRSV-2	
1	PRRSV/MUT46	+	−	+	100
2	PRRSV/MUT47	+	+	−	100
3	PRRSV/MUT49	+	+	+	100
4	PRRSV/MUT50	+	+	+	100
5	PRRSV/MUT53	+	−	−	N/A
6	PRRSV/MUT56	+	−	+	100
7	PRRSV/MUT57	+	+	+	100
8	PRRSV/MUT58	+	−	−	N/A
9	PRRSV/MUT59	+	+	+	100
10	PRRSV/MUT60	+	+	−	100
11	PRRSV/MUT61	+	−	+	100
12	PRRSV/MUT62	+	−	+	100
13	PRRSV/MUT64	+	−	+	100
14	PRRSV/MUT65	+	−	+	100
15	PRRSV/MUT68	+	−	+	100
16	PRRSV/MUT70	+	−	+	100
17	PRRSV/MUT77	+	−	+	100
18	PRRSV/MUT79	+	+	−	100
19	PRRSV/MUT80	+	−	+	100
20	PRRSV/MUT87	+	−	+	100
21	PRRSV/MUT88	+	−	+	100
22	PRRSV/MUT89	+	−	-	N/A
23	PRRSV/MUT90	+	−	+	100
24	PRRSV/MUT96	+	−	+	100

a First-round nested RT-PCR using an external primer to detect PRRSV-1 (637 bp) and PRRSV-2 (660 bp);

b Second-round nested RT-PCR using internal primers to detect PRRSV-1 (158 bp) and PRRSV-2 (287 bp); N/A = Data not available for real-time RT-PCR, + = Positive results, − = Negative results, RT-PCR = Reverse transcription polymerase chain reaction, PRRSV = Porcine reproductive and respiratory syndrome virus, ORF = Open reading frame, MUT46 = Sample ID used in this study

### Sequencing and phylogenetic confirmation of PRRSV genotypes

**Figure 4:**
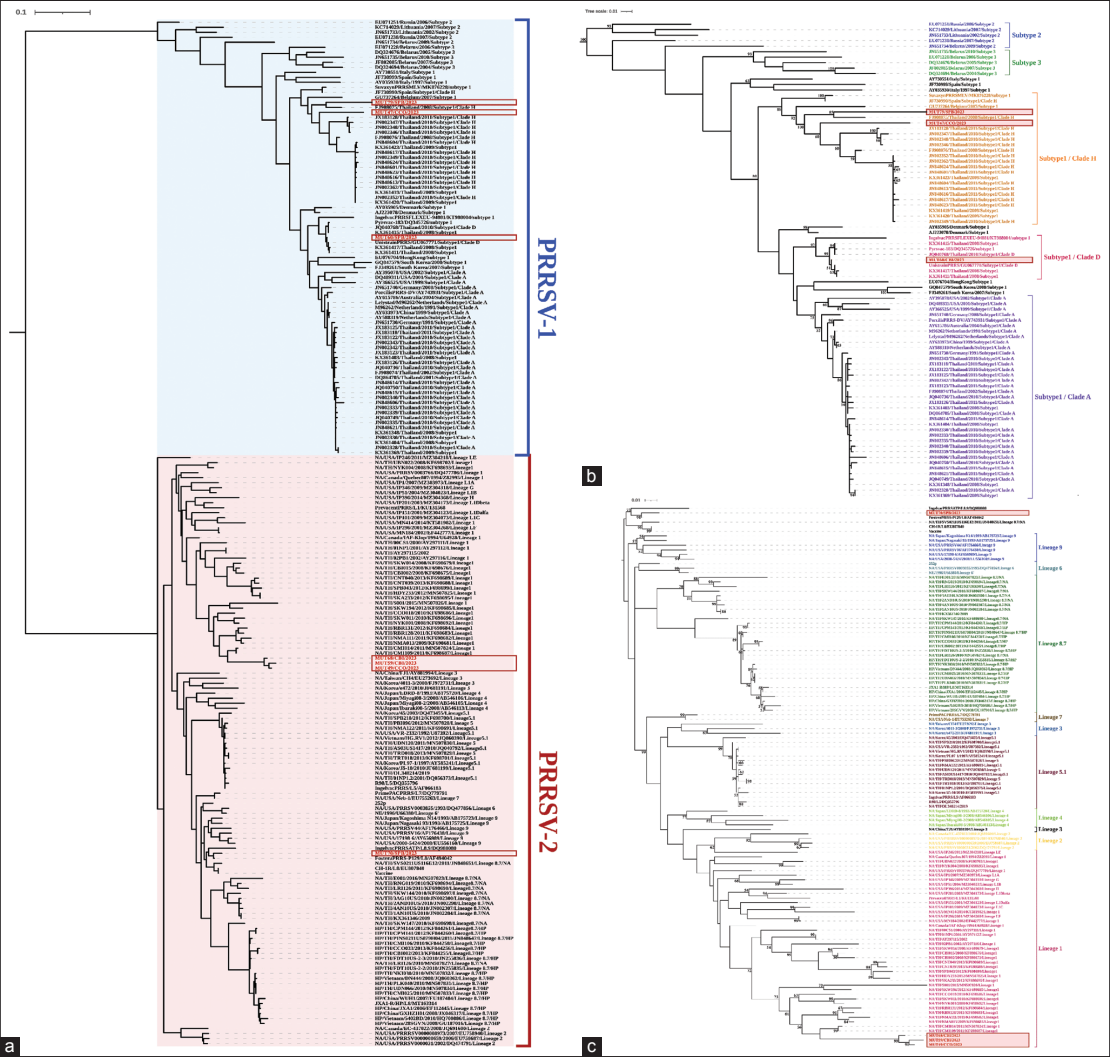
Phylogenetic analysis of PRRSV strains based on ORF5 nucleotide sequences, performed using the MEGA X software’s maximum likelihood method. (a) Phylogenetic tree constructed from a total of 205 PRRSV sequences, including both PRRSV-1 and PRRSV-2 strains. (b) Phylogenetic tree derived from 87 PRRSV-1 sequences. (c) Phylogenetic tree derived from 118 PRRSV-2 sequences. Only bootstrap values greater than or equal to 50% are displayed in the tree branch. PRRSV = Porcine reproductive and respiratory syndrome virus, ORF = Open reading frame.

The ORF5 gene was amplified and sequenced from seven representative positive samples to confirm PRRSV genotypes. These included three PRRSV-1 strains (PRRSV/MUT47, PRRSV/MUT60, PRRSV/MUT79) and four PRRSV-2 strains (PRRSV/MUT49, PRRSV/MUT59, PRRSV/MUT68, PRRSV/MUT70). Although PRRSV/MUT49 and PRRSV/MUT59 tested positive for both PRRSV-1 and PRRSV-2, the concentration of PRRSV-1 PCR products was <25 ng/μL, which was insufficient for sequencing. The sequences were compared with GenBank entries using BLAST, showing high similarity to reference strains (Table 5). For PRRSV-1, MUT47 was closely related to EU/TH/CBI025/2013 (subtype 1, clade H, Thailand), MUT60 to PRRSV1/Pig-wt/CAT-Esp/N6b/May-2021 (Spain, 2021), and MUT79 to EU/TH/SKA060/2010 (subtype 1, clade H, Thailand). For PRRSV-2, MUT49, MUT59, and MUT68 matched NA/TH/CMI109/2011 (lineage 1, Thailand), whereas MUT70 was closely related to P129 Fostera PRRS (lineage 8). Phylogenetic analysis based on 205 global ORF5 sequences (Shi *et al*. [10]) clearly separated PRRSV-1 and PRRSV-2 (Figure 4a). The Thai PRRSV-1 strains clustered into subtype 1 clades D and H (Figure 4b), while PRRSV-2 strains were grouped into lineages 1 and 8 (Figure 4c).

**Table 5:** ORF5 nucleotide similarity analysis of Thai PRRSV isolates compared with reference sequences in the GenBank database and phylogenetic analysis to confirm the genetic lineage classification of PRRSV.

Thai PRRSVs	Closest reference sequences	Percentage of nucleotide identity (%)	Phylogenetic analysis
PRRSV/MUT47 (PV440586)	KF698631	92.5	Subtype 1/Clade H
PRRSV/MUT60 (PV440587)	OP822961	100	Subtype 1/Clade D
PRRSV/MUT79 (PV440588)	KF698640	92.24	Subtype 1/Clade H
PRRSV/MUT49 (PV440589)	KF698687	91.04	Lineage 1
PRRSV/MUT59 (PV440590)	KF698687	91.05	Lineage 1
PRRSV/MUT68 (PV440591)	KF698687	91.04	Lineage 1
PRRSV/MUT70 (PV440592)	AF494042	98.83	Lineage 8

PRRSV = Porcine reproductive and respiratory syndrome virus, ORF = Open reading frame, MUT47 = Sample ID used in this study

### Genetic classification of Thai PRRSV-2 strains based on global ORF5 sequences

**Figure 5:**
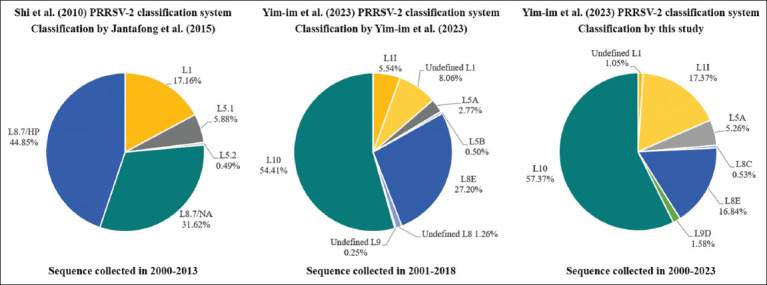
Phylogenetic classification of Thai PRRSV-2 isolates based on different genetic classification systems. Pie charts illustrate the proportion of PRRSV-2 lineages across different time periods, categorized by three classification methods. The Shi *et al*. [10] system, as applied by Jantafong *et al*. [21] to sequences collected from 2000–2013, shows a predominance of L8.7/HP and L8.7/NA. The Yim-im *et al*. [13] system indicates L10 as the dominant lineage in the 2001–2018 datasets, followed by L8E. Classification of sequences collected in the present study (2000–2023) using the Yim-im *et al*. [13] system confirms L10 as the predominant lineage, with L8E as the second most common. Notably, this study also identified newly emerging sublineages, L8C and L9D, which had not been previously reported in Thailand. PRRSV = Porcine reproductive and respiratory syndrome virus.

**Figure 6:**
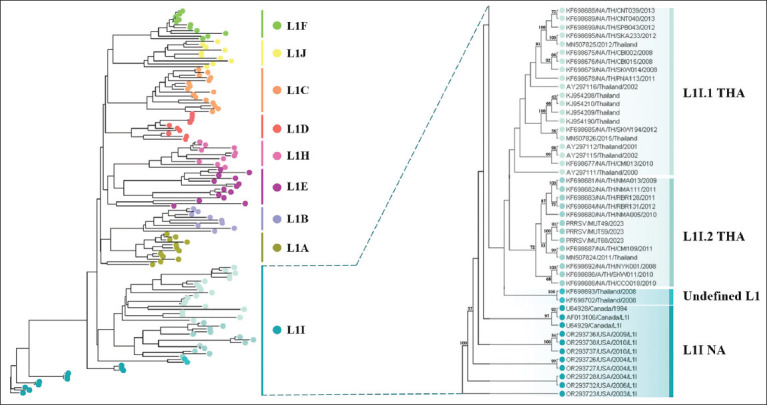
Independent phylogenetic analysis of 138 ORF5 gene sequences of PRRSV-2 strains belonging to lineage L1 to elucidate sublineage diversity in the context of Thailand. The phylogenetic tree was constructed using complete ORF5 nucleotide sequences retrieved from the GenBank database and classified into L1A–L1F and L1H–L1J sublineages. Only bootstrap values greater than or equal to 50% are displayed in the tree branch. PRRSV = Porcine reproductive and respiratory syndrome virus, ORF = Open reading frame.

To further refine the genetic classification of Thai PRRSV-2 between 2000 and 2023, phylogenetic analysis was performed using the global PRRSV-2 classification system proposed by Yim-im *et al*. [13], which is based on 82,237 ORF5 sequences. A total of 386 complete PRRSV-2 ORF5 sequences were analyzed, including 190 Thai isolates and 196 references. The Thai strains were assigned to seven lineages: undefined L1 (n = 2, 1.05%), L1I (n = 33, 17.37%), L5A (n = 10, 5.26%), L8C (n = 1, 0.53%), L8E (n = 32, 16.84%), L9D (n = 3, 1.58%), and L10 (n = 109, 57.37%) (Figure 5 [10, 13, 21] and supplementary Figure 1). A more detailed evaluation of the 138 lineage L1 sequences showed that 35 Thai strains belonged to undefined L1 (n = 2) and L1I (n = 33), with no evidence of L1A–L1F, L1H, or L1J sublineages in Thailand. L1I emerged as the dominant sublineage in Thai swine herds. Within L1I, three evolutionary branches were identified: L1I-NA (North America), L1I.1-THA (Thailand, 2000–2015), and L1I.2-THA (Thailand, 2008–2023) (Figure 6).

## DISCUSSION

The swine industry plays a vital role in animal protein production in Thailand due to the country’s high pork consumption, making it a cornerstone of the national economy. However, this sector faces considerable challenges from harmful pathogens, particularly the economically significant PRRSV [23]. At present, Thailand is experiencing endemic PRRS primarily caused by PRRSV-1 and PRRSV-2 infections. Although PRRSV-2 remains the dominant strain circulating in the country, cases of mixed infections with both genotypes have been documented in swine farms [13, 15, 19, 21], complicating disease control and management strategies.

### Establishment of nested ORF7 RT-PCR for PRRSV detection and differentiation

Despite the availability of diverse diagnostic methods for PRRSV, challenges in early detection and disease prevention remain [16]. Various molecular techniques, including RT-PCR, qRT-PCR, and LAMP, are highly effective for rapid screening and accurate detection; however, each has limitations. The sensitivity of RT-PCR decreases with low viral load samples [24]. LAMP assays can generate false positives due to primer dimer formation [16, 25], while TaqMan probe-based qRT-PCR, though highly specific, is often too costly for small-scale applications [16, 26]. Advanced methods such as SYBR Green I-based duplex qRT-PCR enable precise and rapid genotyping but require skilled personnel and advanced laboratory infrastructure [26]. DNA sequencing is considered the gold standard for genotyping, and mNGS allows unbiased detection of variant strains [16, 27]; however, both are constrained by high costs and technical expertise requirements, limiting their use in resource-limited laboratories. Thus, alternative molecular assays that combine high specificity with operational simplicity are urgently needed.

Nested PCR, an in vitro gene amplification technique, was developed to improve the sensitivity and specificity of conventional PCR. Unlike standard PCR, it employs two sets of primers: an outer primer and an inner primer [28]. The first primer set amplifies the target fragment in a conventional PCR reaction. The second set of primers, known as nested primers, then binds to the initial PCR product, producing a shorter secondary amplicon [29]. This method enhances assay sensitivity by re-amplifying the target sequence while avoiding re-amplification of non-target sequences generated in the first round. Consequently, nested PCR significantly improves both sensitivity and specificity, enabling detection of target DNA at much lower concentrations compared with conventional PCR [30].

In this study, we developed a nested ORF7 RT-PCR assay to improve PRRSV detection and differentiation. By targeting the relatively conserved ORF7 region, which encodes the N protein, the assay provides a reliable and efficient tool for species-specific differentiation. The ORF7 region was selected due to its high expression during viral replication and its sufficient sequence divergence between PRRSV-1 and PRRSV-2, making it an ideal target for genotyping. This method offers a practical and straightforward alternative to costly qRT-PCR platforms for resource-limited laboratories.

To optimize the detection and differentiation of PRRSV genotypes, reaction conditions were systematically refined. The reaction mixture was precisely formulated to contain 10 pM primers, 10 mM dNTPs, and 1.0 U of Platinum *Taq* DNA polymerase. Optimization of PCR components is critical for successful amplification as it directly impacts assay efficiency and specificity [31]. Therefore, all components were comprehensively evaluated to maximize sensitivity, specificity, and overall assay performance, while minimizing non-specific products and artifacts [32]. Specificity testing was further validated using vaccine strains, clinically positive samples, and dual-infected samples, yielding consistent and reproducible results with distinct bands corresponding to each target. These findings demonstrate the assay’s robustness in identifying individual PRRSV strains, even in mixed infections, underscoring its diagnostic applicability.

### Diagnostic accuracy of nested ORF7 RT-PCR

Following application in clinical samples, the protocol was refined to address issues of non-specific amplification in pigs infected with multiple strains. Adjustments to annealing times improved product yield during the second PCR round, accounting for the relatively small amplicon sizes: 158 bp for PRRSV-1 and 287 bp for PRRSV-2. Each reaction was performed independently with specific primers, and cycling conditions were optimized to ensure consistency across both reactions. This refinement reduced reaction time in the second step, providing a highly efficient and specific protocol for detecting and differentiating PRRSV-1 and PRRSV-2.

Compared with earlier nested RT-PCR methods [19, 33–35], which required lengthy processing to obtain results, the modified protocol offers faster and more reliable detection. Although our method provides clear advantages in efficiency and simplicity, it is important to note that RT-qPCR has become the preferred tool for routine diagnostics due to its speed, sensitivity, and ability to multiplex strains [36–38].

At present, real-time RT-PCR is widely regarded as the gold standard for PRRSV diagnosis [39]. It is extensively employed for clinical detection due to its high sensitivity, specificity, rapid turnaround time, and quantitative capabilities [40–42]. Nonetheless, it requires expensive equipment and specialized training [17, 39], and it cannot generate complete sequence data for phylogenetic analysis, limiting its utility for subgroup or lineage identification. In this study, the nested ORF7 RT-PCR showed 100% concordance with real-time RT-PCR in clinically positive samples, confirming its reliability for distinguishing PRRSV-1 and PRRSV-2. Furthermore, the first-round PCR amplifies the full ORF7 gene, providing valuable material for molecular characterization and genetic diversity studies. Collectively, the improved specificity, efficiency, and reliability of this assay highlight its broad applicability in both diagnostics and genetic monitoring of PRRSV, particularly in resource-limited regions.

### ORF5 sequencing for confirmation of PRRSV genotypes

Although ORF7 encodes the most abundant structural protein of PRRSV and is highly immunogenic [43], it has limited resolution for phylogenetic analysis. The ORF5 gene, by contrast, is more genetically variable and widely used for global PRRSV classification, making it better suited for lineage differentiation [25, 44].

In this study, sequencing of the ORF5 gene from seven clinical samples enabled precise genotype confirmation. Among PRRSV-1 strains, PRRSV/MUT47 and PRRSV/MUT79 closely resembled a Thai PRRSV-1 subtype 1/clade H strain [21], indicating ongoing local circulation. PRRSV/MUT60 clustered with a subtype 1/clade D strain from Spain, suggesting possible introduction of foreign strains through trade or animal movement. For PRRSV-2, strains PRRSV/MUT49, PRRSV/MUT59, and PRRSV/MUT68 were highly similar to lineage 1 strain NA/TH/CMI109/2011, reflecting the persistence of endemic strains. Interestingly, PRRSV/MUT70 exhibited 98% nucleotide identity with the prototype strain of the Fostera PRRS MLV vaccine (P129, lineage 8). According to ORF5 sequence identity criteria, strains with >97% nucleotide similarity are classified as the same [45], suggesting that PRRSV/MUT70 may be vaccine-derived. Given that modified live vaccines can replicate in hosts [46], confirmation of vaccine-derived origin is recommended.

The detection of diverse PRRSV lineages within a small sample set highlights the genetic variability and dynamic evolution of PRRSV in Thai herds. The coexistence of endemic, imported, and potentially vaccine-related strains has significant implications for vaccination strategies, biosecurity, and disease control [47]. By integrating molecular diagnostics with sequence-based classification, this study enhances understanding of PRRSV genetic diversity in Thailand.

### Refining the genetic classification of Thai PRRSV-2 strains

PRRSV continues to pose a major threat to swine populations in Thailand despite extensive control efforts, and its complete eradication remains elusive. A key challenge lies in its high genetic diversity, which accelerates the emergence of new viral strains and undermines vaccine efficacy. Therefore, continuous genetic characterization and analysis within a globally recognized classification framework are essential.

In 2010, a comprehensive phylogenetic analysis of 8,624 ORF5 sequences from field isolates and vaccine strains worldwide established a systematic classification of PRRSV-2 using Bayesian methods [10]. This study divided PRRSV-2 into nine monophyletic lineages (L1–L9) and further subdivided into 37 sublineages (e.g., L1: 1.1–1.9; L5: 5.1–5.2; L8: 8.1–8.9; L9: 9.1–9.17). Importantly, more than 85% of isolates clustered into lineages 1, 5, 8, and 9. In Thailand, surveillance studies identified the predominance of lineages 1, 5.1, 5.2, and 8.7. Sublineage 8.7, in particular, encompassed two distinct groups: 8.7/HP and 8.7/NA. The 8.7/HP strains were further divided into clades A (SX2009-like), B (09HEN1-like), and JXA1-like isolates [21]. However, since 2015, the phylogenetic classification of PRRSV-2 in Thai swine populations has not been updated. Recently, Yim-im *et al*. [13] refined the classification of PRRSV-2 (1998–2021), providing an updated framework for global genetic diversity analysis.

In this study, a maximum likelihood (ML) phylogenetic tree was constructed based on ORF5 sequences following the system proposed by Yim-im *et al*. [13]. The dataset included all PRRSV-2 ORF5 sequences available from Thailand between 2000 and 2023, from which 190 carefully curated sequences were selected for analysis. This approach categorized Thai PRRSV-2 strains into five major lineages (L1, L5, L8, L9, and L10) and five sublineages (L1I, L5A, L8C, L8E, and L9D), thereby refining earlier classification efforts.

Compared with previous studies by Jantafong *et al*. [21] and Yim-im *et al*. [13], the findings revealed both similarities and differences. While Jantafong *et al*. [21] identified sublineage L8E (formerly 8.7/HP) as dominant between 2000 and 2013, the current analysis indicates a shift toward lineage L10 (historically L8.7/NA) in more recent years. This aligns with Yim-im *et al*. [13], who also reported L10 as the prevailing lineage between 2001 and 2018. In addition, this study provides the first evidence of L8C and L9D sublineages in Thailand, expanding the known genetic landscape.

The rise of L10 as the dominant epidemic variant suggests dynamic evolutionary shifts influenced by host immunity, vaccine pressure, viral fitness, and changes in production systems. The detection of L8C and L9D highlights the need for continuous molecular surveillance and lineage monitoring to detect novel or re-emerging strains that may complicate control efforts.

### Lineage 1 (L1): Global prevalence and Thai emergence

Lineage 1 (L1) is among the most genetically diverse and globally widespread PRRSV-2 lineages, consisting of nine sublineages (L1A–L1F, L1H–L1J) [48]. Its success is attributed to persistent transmission, high mutation rates, and rapid adaptation to immune and environmental pressures [12], which complicate disease control and necessitate ongoing surveillance.

Globally, L1A and L1C sublineages have predominated, particularly in the United States and China, gradually replacing formerly dominant lineages such as L8 [13, 49]. In Thailand, earlier studies reported L10 as the prevailing genotype during 2009–2013, with sporadic detections until 2016. However, more recent molecular investigations, including those by Yim-im *et al*. [13] and the present study, have documented the emergence and increasing prevalence of the L1I sublineage between 2015 and 2023. Representative L1I strains identified here include NA/TH/S001/2015 (MN507826), MUT49/CCO/2023 (PV440589), MUT59/CBI/2023 (PV440590), and MUT68/CBI/2023 (PV440591).

The detection of NA/TH/S001/2015 during severe PRRS outbreaks in vaccinated herds underscores the significance of immune escape variants. This strain shares <87% amino acid identity in the GP5 protein compared with the vaccine prototype strain. Key mutations in epitope A, T-cell epitopes, and N-linked glycosylation sites of GP5 are associated with altered antigenicity and immune evasion [50]. These features likely contribute to vaccine failures and recurrent outbreaks despite immunization.

The rise of L1I as a genetically distinct and immunologically evasive sublineage signals a shift in PRRSV epidemiology in Thailand. It highlights the limitations of current vaccine formulations, which may not adequately protect against divergent L1I strains. Continuous evaluation of vaccine efficacy and adaptation of immunization strategies to include locally relevant genotypes are therefore essential. Collectively, these findings emphasize the critical importance of sustained molecular surveillance and genetic monitoring to inform evidence-based control measures, minimize economic losses, and safeguard animal health.

## CONCLUSION

This study successfully established and optimized a nested ORF7 RT-PCR assay for the detection and differentiation of PRRSV-1 and PRRSV-2 in Thailand. The assay demonstrated high specificity, reproducibility, and 100% concordance with real-time RT-PCR when tested on clinically positive samples. Importantly, amplification of the full ORF7 gene provides an additional advantage for molecular characterization and genetic diversity studies, thereby extending its application beyond routine diagnostics. Sequencing of representative ORF5 genes further confirmed the co-circulation of endemic, imported, and vaccine-derived PRRSV strains, revealing a dynamic evolutionary landscape in Thai swine herds. Notably, the emergence of lineage L10 as the dominant epidemic variant and the detection of L1I sublineages with immune escape potential highlight the ongoing genetic shifts that may undermine current vaccine strategies.

From a practical standpoint, this nested RT-PCR assay represents a cost-effective, operationally simple, and resource-appropriate diagnostic tool, particularly suited for laboratories in low-resource settings where advanced qRT-PCR platforms are unavailable. Its ability to differentiate genotypes in mixed infections makes it valuable for epidemiological surveillance, outbreak investigation, and vaccine policy decision-making.

The strength of this study lies in its integration of molecular assay development with genetic surveillance, providing both a diagnostic solution and insights into the evolutionary dynamics of PRRSV in Thailand. The identification of new sublineages (L8C and L9D) and the increasing prevalence of L1I strains underscore the necessity for continuous molecular monitoring to anticipate and manage emerging threats.

Nevertheless, several limitations remain. Comprehensive diagnostic performance metrics, including sensitivity, specificity, predictive values, and limit of detection, were not fully quantified. In addition, reliance on ORF5 and ORF7 sequences, while informative, does not capture recombination events or broader genomic variation that could influence vaccine efficacy and viral evolution.

Future studies should therefore focus on large-scale validation of the nested assay across diverse field conditions, direct comparison with multiplex qRT-PCR platforms, and whole-genome sequencing of circulating strains to refine classification, identify recombination, and inform the design of next-generation vaccines. Longitudinal studies combining molecular surveillance with immunological and epidemiological data will be critical for developing more effective, region-specific PRRSV control strategies.

In conclusion, the nested ORF7 RT-PCR assay developed in this study offers a practical, reliable, and accessible diagnostic alternative for the detection and differentiation of PRRSV genotypes. Combined with phylogenetic refinement of ORF5 sequences, it provides an integrated framework for advancing PRRSV diagnostics, surveillance, and control in Thailand and other resource-limited settings. By bridging diagnostic innovation with molecular epidemiology, this study contributes to strengthening the One Health approach in managing one of the most economically impactful diseases in the global swine industry.

## DATA AVAILABILITY

The data supporting the findings of this study are available from the corresponding author upon reasonable request.

## AUTHORS’ CONTRIBUTIONS

TJ: Conceptualization, methodology, investigation, data curation, project administration, and drafted the manuscript. SP: Validation, formal analysis, data curation, visualization, and drafted and edited the manuscript. NK, PM, and WT: Formal analysis, visualization, edited the manuscript. All authors have read and approved the published version of the manuscript.
